# The Evolving Role of Fetuin-A in Nonalcoholic Fatty Liver Disease: An Overview from Liver to the Heart

**DOI:** 10.3390/ijms22126627

**Published:** 2021-06-21

**Authors:** Teoman Dogru, Ali Kirik, Hasan Gurel, Ali A. Rizvi, Manfredi Rizzo, Alper Sonmez

**Affiliations:** 1 Department of Gastroenterology, Balikesir University Medical School, Cagis, Balikesir 10145, Turkey; teomandogru@balikesir.edu.tr; 2 Department of Internal Medicine, Balikesir University Medical School, Cagis, Balikesir 10145, Turkey; ali.kirik@balikesir.edu.tr; 3 Department of Gastroenterology, Samsun Education and Research Hospital, University of Health Sciences, Ilkadim, Samsun 55090, Turkey; hasan.gurel1@saglik.gov.tr; 4 Division of Endocrinology, Metabolism, and Lipids, Department of Medicine, Emory University, Atlanta, GA 30322, USA; ali.abbas.rizvi@emory.edu; 5 Division of Endocrinology, Diabetes and Metabolism, Department of Medicine, University of South Carolina, Columbia, SC 29208, USA; manfredi.rizzo@unipa.it; 6 Department of Health Promotion, Mother and Child Care, Internal Medicine and Medical Specialties (PROMISE), University of Palermo, 90133 Palermo, Italy; 7 Department of Endocrinology and Metabolism, Gulhane Medical School, University of Health Sciences, Ankara 06010, Turkey

**Keywords:** NAFLD, Fetuin-A, CVD

## Abstract

Nonalcoholic fatty liver disease (NAFLD) is strongly associated to the features of metabolic syndrome which can progress to cirrhosis, liver failure and hepatocellular carcinoma. However, the most common cause of mortality in people with NAFLD is not liver-related but stems from atherosclerotic cardiovascular disease (CVD). The prevalence of NAFLD is on the rise, mainly as a consequence of its close association with two major worldwide epidemics, obesity and type 2 diabetes (T2D). The exact pathogenesis of NAFLD and especially the mechanisms leading to disease progression and CVD have not been completely elucidated. Human fetuin-A (alpha-2-Heremans Schmid glycoprotein), a glycoprotein produced by the liver and abundantly secreted into the circulation appears to play a role in insulin resistance, metabolic syndrome and inflammation. This review discusses the links between NAFLD and CVD by specifically focusing on fetuin-A’s function in the pathogenesis of NAFLD and atherosclerotic CVD.

## 1. Introduction

NAFLD is the most common liver disorder, affecting 30–40% of the adult population [1,2,3] and up to 95% of patients with obesity [4,5,6]. It is characterized by chronic accumulation of fat in the liver (>5% of hepatocytes by histology) in the absence of substantial alcohol consumption or other causes of liver disease such as viral or autoimmune hepatitis and medications. It refers to a spectrum of disorders ranging from benign simple steatosis (SS) to potentially more rapidly progressive histological phenotype, such as nonalcoholic steatohepatitis (NASH), which can progress to advanced fibrosis, cirrhosis and hepatocellular carcinoma [7,8,9]. A large body of evidence indicates that NAFLD is closely related to metabolic syndrome (MetS), and this association is mutual and bi-directional [10]. Insulin resistance seems to play a key role in the pathogenesis of NAFLD [11,12,13]. The pathophysiologic mechanisms underlying NAFLD and its progression have not been completely elucidated so far. The interplay between the delivery of lipids to the liver and the processes of hepatic lipid uptake, synthesis, oxidation and secretion is modified by the hepatokines, which are proteins secreted by the liver. The alterations in the secretion and phosphorylation of hepatokines play a role in the pathogenesis of NAFLD [14,15]. CVD is the most common cause of mortality in patients with NAFLD [16,17]. The focus of this article is to explore the relation of NAFLD to increased CVD risk. 

A growing body of evidence suggests that NAFLD increases the risk of CVD, although the exact pathogenesis is not clear [18,19,20,21]. Whether this is an independent effect of NAFLD or is confounded by the shared risk factors of insulin resistance, diabetes mellitus, dyslipidemia or hypertension is not clearly identified [16,17]. Prospective cohort studies performed in patients with T2D reported the role of NAFLD as an independent predictor of the future risk of incident CVD [22,23]. On the other hand, several studies show that atherogenic dyslipidemia, hyperinsulinemia and impaired liver glucose output are the possible players in the interaction between NAFLD and CVD [24,25,26]. So far, the pathogenesis of CVD in people with NAFLD is not clear and the search for the missing link is still in progress.

**Figure 1:**
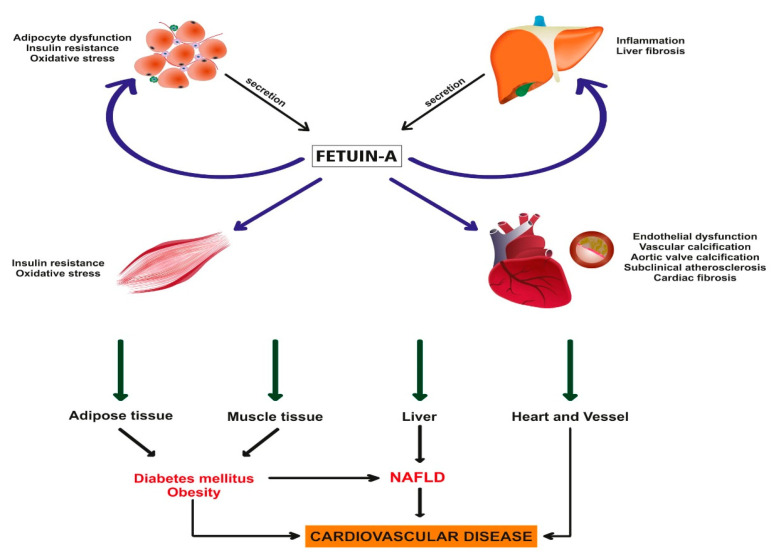
The pathophysiological relationship of fetuin-A with NAFLD and CVD. Increased fetuin-A in plasma exerts an insulin pathway inhibitory effect by modulating the kinase reaction on the insulin-receptor tyrosine kinase; it leads to the development of insulin resistance in insulin-sensitive tissues (muscle tissue, adipose tissue, liver, etc.) Furthermore, fetuin-A stimulates the release of inflammatory cytokines through Toll-like receptor 4 (TLR4) and it causes systemic inflammation. On the other hand, fetuin-A is an important calcium- and phosphate-binding protein and decreased fetuin-A levels strongly correlate with excessive vascular calcification and heart failure. As a result, fetuin-A protein shows a multifunctional effect in the MetS and it plays a key role in the pathogenesis of NAFLD, T2D, obesity and CVD.

Fetuin-A, also known as alpha-2-Heremans-Schmid glycoprotein, is abundantly synthesized and secreted by the liver. It is found in the extracellular space throughout the body [27]. Fetuin-A is a multifaceted protein playing a role in various cellular pathways including calcium and bone metabolism, insulin resistance and inflammation. The genes encoding fetuin-A are involved in diseases like MetS and T2D [15]. It is an important inhibitor molecule of the insulin receptor tyrosine kinase in studies of insulin-resistant animal model [28,29,30]. In addition, fetuin-A is thought to support the formation of insulin resistance with its proinflammatory effect, apart from its direct effect on the insulin receptor [31]. It is thought that fetuin-A, which has a direct effect on insulin resistance, modulates for inflammatory reactions and causes various metabolic alterations [32]. Consistent with these findings, many epidemiologic studies showed that higher serum fetuin-A concentrations were independently associated with T2D, insulin resistance, MetS and CVD [33,34,35]. On the other hand, there are limited and conflicting data about the relationship between fetuin-A and NAFLD. The current review focuses on the role of fetuin-A in the pathogenesis of NAFLD and NAFLD-associated CVD risk (Figure 1).

## 2. The Role of Fetuin-A in NAFLD; from Hepatic Steatosis to Inflammation and Fibrosis

NAFLD occurs due to increased uptake and deposition of lipids in the hepatocytes which surpasses the rate of mitochondrial fatty acid oxidation and triglyceride secretion as the very low density lipoprotein particles. The disruption of the balance between lipid uptake and secretion is related to the impaired synthesis and secretion of hepatokines, leading to the development of insulin resistance, glucose intolerance and inflammation [14]. Fetuin-A, the major protein of the alpha-2-globulin fraction in serum electrophoresis, is among the hepatokines highly related to the pathogenesis of NAFLD and its metabolic complications [15]. Fetuin-A expression is significantly increased in subjects with NAFLD [35,36] and decrease after the improvement of NAFLD [37]. However, there are conflicting data regarding the relationship of circulating fetuin-A with NAFLD and other metabolic disorders.

### 2.1. Studies Investigating Fetuin-A in Subjects with Radiologically Diagnosed NAFLD

In a prospective study, Lebensztejn et al. investigated circulating fetuin-A levels in 45 obese children with NAFLD diagnosed with abdominal ultrasonography (US). Serum fetuin-A concentration was significantly higher in patients with NAFLD when compared to 30 healthy controls. However, there was no association of fetuin-A with any other parameters studied both in children with NAFLD and obese children without NAFLD [38]. Reinehr et al. studied the relationships between fetuin-A, NAFLD and MetS in a total of 36 obese and 14 lean children. The 12 obese children with NAFLD had significantly higher fetuin-A levels than the 24 obese children without NAFLD and the 14 normal-weight children. Fetuin-A levels were independent of age, pubertal stage and gender. Fetuin-A correlated significantly with systolic and diastolic blood pressure, homeostasis model assessment for insulin resistance (HOMA-IR) and high-density lipoprotein cholesterol (HDL-C) [39]. In a cross-sectional case-control study, Ou HY et al. investigated the relationship between serum fetuin-A levels and prediabetes in subjects with or without ultrasound-diagnosed NAFLD. A total of 510 age- and sex-matched subjects with normal glucose tolerance (NGT), impaired fasting glucose (IFG) and impaired glucose tolerance (IGT) with or without NAFLD were recruited. Fetuin-A levels were significantly higher in subjects with NAFLD when compared to subjects with NGT and prediabetes. Fetuin-A levels were positively associated with postload 2 h glucose, body mass index (BMI), triglyceride and HOMA-IR but negatively associated with age, HDL-C and adiponectin [40]. In a population-based cross-sectional study, Huang et al. investigated the association of serum fetuin-A with fatty liver index (FLI), the indicator of NAFLD in a total of 5219 middle-aged and elderly participants. Fetuin-A was positively associated with FLI, alanine aminotransferase (ALT), aspartate aminotransferease (AST), and gamma-glutamyl transferase (GGT) after adjustment for the confounding factors. Multivariate logistic regression analysis showed that each one-standard-deviation increase in fetuin-A level was associated with 12%, 13%, and 10% increased risk of elevated FLI, ALT, and AST, respectively. Categorical analysis showed that compared to the lowest quartile, the highest quartile of serum fetuin-A was associated with a 35%, 50% and 33% increased risk of elevated FLI, ALT and AST, respectively [41]. Thompson et al. investigated the relationship of blood fetuin-A with fatty liver in 78 nonobese persons of African origin. Liver and abdominal fat were evaluated using computed tomography (CT). No association was found between fetuin-A and liver fat content [42]. Cui et al. investigated the association of serum fetuin-A with NAFLD in 79 Chinese subjects. NAFLD was diagnosed and graded based on abdominal US. Serum fetuin-A level in NAFLD patients was significantly lower than that the controls. In addition, compared with controls, mild NAFLD and moderate NAFLD had significantly lower concentration of fetuin-A, while fetuin-A level tended to increase slightly with the severity of NAFLD [43]. Studies investigating fetuin-A in subjects with radiologically diagnosed NAFLD are given in Table 1.

### 2.2. Studies Investigating Fetuin-A in Histologically Diagnosed Subjects with NAFLD

Yilmaz et al. investigated circulating fetuin-A in 99 patients with biopsy-proven NAFLD and 75 age- and gender-matched healthy controls. Fetuin-A levels were significantly higher in subjects with NAFLD when compared to the controls. Multivariate analysis revealed a significant association of fetuin-A with insulin resistance as assessed by the HOMA-IR and the liver fibrosis. Moreover, the relationship between fetuin-A and fibrosis remained statistically significant even after adjustment for potential confounders, including the insulin resistance [45]. In a randomized controlled trial, Haukeland et al. investigated 111 subjects with histologically proven NAFLD. One hundred and thirty-one healthy subjects served as healthy controls. The main outcome variables were circulating levels of fetuin-A according to the presence of NAFLD, hepatic gene expression of fetuin-A and key enzymes in glucose and lipid metabolism. Fetuin-A levels were significantly higher in patients with NAFLD compared to controls. NAFLD was a significant predictor of elevated fetuin-A independent of BMI, age, sex, fasting glucose and triglycerides. Hepatic fetuin-A mRNA levels correlated significantly with hepatic mRNA levels of key enzymes in lipid (sterol regulatory element-binding protein-1c, carnitine palmitoyltransferase-1) and glucose (phosphoenol pyruvate kinase-1, glucose-6-phosphatase) metabolism [46]. Ou et al. aimed to investigate the levels of fetuin-A in 180 age- and sex-matched subjects with NGT, NAFLD, newly diagnosed T2D (NDT2D) and NDT2D with NAFLD. They observed that fetuin-A levels were significantly higher in NDT2D with NAFLD as compared with NDT2D or NAFLD subjects [47]. In our previous study, we investigated circulating concentrations of fetuin-A and its possible association with hepatic and systemic inflammation in a total of 105 nondiabetic male subjects with biopsy-proven NAFLD (NASH, n = 86 and SS, n = 19). Plasma levels of fetuin-A and markers of inflammation [high-sensitivity C-reactive protein (hsCRP), tumor necrosis factor alpha (TNF-α), interleukin-6 (IL-6) and adiponectin] were determined. In multivariate analysis, fetuin-A was negatively correlated with age, however, there was no association between fetuin-A and BMI, waist circumference (WC), glucose, insulin, HOMA-IR, lipid parameters, and inflammatory markers. In addition, no significant association was observed between fetuin-A and histological findings including liver fibrosis [48]. Kahraman et al. examined the hepatic fetuin-A expression in 108 morbidly obese NAFLD patients (50 with NASH and 58 with SS) undergoing bariatric surgery. A total of 10 subjects were used as healthy controls. In addition, primary human hepatocytes were treated with non-esterified fatty acid (NEFA) to investigate changes in fetuin-A expression. Treatment of hepatocytes with NEFA led to up-regulation of fetuin-A expression. Fetuin-A serum concentrations were not different between NAFLD patients (or subgroups) and controls. In liver tissue, expression of fetuin-A was significantly elevated in SS compared with controls. The NASH group exhibited an even stronger increased mRNA expression than SS patients. In correlation analysis, a significant positive association of fetuin-A with liver mRNA was observed. In addition, a significant negative association was found for the fibrosis stage and serum fetuin-A [49]. In a cross-sectional study, Rametta et al. evaluated the causal relationship between fatty liver and serum fetuin-A levels in 137 patients with histologically diagnosed NAFLD and 260 healthy subjects. They also analyzed whether the inherited PNPLA3 I148M variant modulates fetuin-A. Fetuin-A levels were higher in NAFLD patients than in the controls, independently of age, sex, BMI, insulin resistance, dyslipidemia, adiponectin, PNPLA3 I148M and ALT levels. In NAFLD patients, fetuin-A was associated with steatosis severity and MetS, but not with hepatic inflammation. At multivariate analysis, fetuin-A levels were associated with BMI, triglycerides, hyperglycemia and PNPLA3 I148M independently also of age, sex and ALT levels [50]. In their study, Von Loeffelholz et al. studied the role of fetuin-A in 58 patients with NAFLD/NASH undergoing open abdominal surgery. Compared to non-NAFLD subjects, fetuin-A levels were found to be significantly increased in subjects with NAFLD and NASH. In addition, fetuin-A correlated with the extent liver steatosis and hepatocellular ballooning degeneration [51]. However, on multivariate analysis, only hepatic steatosis was related with fetuin-A. In their study, Peter et al. reported in 55 NAFLD subjects, hepatic mRNA expression of fetuin-A associated positively with liver triglyceride content and HOMA-IR. In 220 subjects who underwent precise metabolic phenotyping, circulating fetuin-A was associated positively with liver fat content, and negatively with insulin sensitivity measured with the oral glucose tolerance test (OGTT) and during the euglycemic, hyperinsulinemic clamp [53]. Pampanini et al. studied fetuin-A levels in 81 obese children with NAFLD diagnosed by biopsy, 79 obese children with NAFLD defined by liver US and 23 lean subjects. Obese children with NAFLD detected by US had significantly higher fetuin-A levels compared to those with normal liver. In obese children who underwent liver biopsy, no significant differences were detected in fetuin-A levels between subject with NASH and those with SS [52]. Studies investigating fetuin-A in histologically diagnosed NAFLD are listed in Table 1.

It is clear that there are significant differences regarding the relationship of circulating fetuin-A with NAFLD in literature. We suggest some possible explanations for the conflicting findings of these studies. First, when the abovementioned studies were analyzed separately, it can be seen that some of the subjects with NAFLD had metabolic confounders such as morbid obesity, T2D, hypertension and MetS. It has been reported that circulating levels of fetuin-A may be easily affected by these metabolic risk factors [31,46]. In addition, some of these patients were also using medications related to these metabolic problems [49,54]. It is well known that certain agents such as metformin, pioglitazone and niacin may affect the fetuin-A concentrations. Therefore, data regarding the association of fetuin-A with NAFLD might be affected by these confounders. Liver biopsy is the gold standard in diagnosing NAFLD, and the most accurate tool for the determination and grading of inflammation and fibrosis. However, in the abovementioned studies, the diagnosis of NAFLD was mostly performed by US or CT, and not all subjects received a liver biopsy. Therefore, the use of the various techniques for diagnosing of NAFLD may also contribute to contradictory results regarding the association of fetuin-A with liver inflammation and fibrosis. Circulating fetuin-A has been inversely associated with age in subjects with NAFLD. Although it has been hypothesized that low levels of fetuin-A may be the result of decreased hepatic fetuin-A production in older people, the exact mechanism responsible for this relationship remains unclear [44,47,48]. Thus, age may be another factor that influences the levels of circulating fetuin-A in NAFLD. The different methods of measuring the concentrations of circulating fetuin-A (such as different ELISA kits) might be another reason for discrepant findings in various studies [45,48,49]. Another reason might be the polymorphisms of the fetuin-A gene which cause variations in the serum assays [55]. On the other hand, as recently reported, genetic factors may also affect the fetuin-A concentrations independently from inflammation [53]. Lastly, other possible confounders that may affect the association of fetuin-A with NAFLD might include arbitrary definitions of “apparently healthy” controls and different definitions regarding the “normal levels of serum aminotransferases”. In light of these data, we think that it is still uncertain whether fetuin-A directly contributes to the development of NAFLD, whether elevated blood levels reflect the presence or severity of NAFLD, or if other unidentified factors simultaneously influence both. Going forward, research should investigate whether fetuin-A has a role in the progression of liver inflammation and fibrosis that may occur during the natural history of NAFLD. Further studies are also needed to search the modulation of fetuin-A as a potential therapeutic strategy in this clinically relevant condition.

## 3. The Relationship of Fetuin-A with NAFLD Associated CVD Risk

The relationship of fetuin-A with CVD is Janus-faced. Both low and high fetuin-A levels have been reported to increase the risk of CVD-related mortality and morbidity [15]. Fetuin-A is a significant regulator of calcium, phosphate and bone metabolism, and a prominent inhibitor of extraosseous calcification [56,57]. Low serum fetuin-A levels are found in patients with chronic kidney disease (CKD), which in turn is associated with arterial stiffness and increased all-cause and cardiovascular mortality [58,59]. On the other hand, high fetuin-A levels may also increase the risk of CVD through effects on insulin resistance and subclinical inflammation [15]. As NAFLD is a significant risk factor for the development of MetS and T2D [60], the relation of fetuin-A to CVD is likely to be stronger in the presence of NAFLD [61].

Few studies have investigated the association of circulating fetuin-A levels and risk of CVD prospectively. In a study of 3810 individuals older than 65 years, it was reported that higher fetuin-A levels were associated with lower risk of CVD among participants without T2D [62]. These findings are similar to the previous Rancho Bernardo Study that showed higher fetuin-A levels were associated with lower risk of CVD mortality in participants without T2D [63]. On the other hand, in the Nurses’ Health Study, higher fetuin-A levels were associated with lower CVD risk when CRP levels were high, but no association was observed among participants with lower CRP levels [64]. Moreover, we and others have demonstrated that fetuin-A level is inversely associated with calcification scores, CV events and mortality from CVD in subjects with CKD [65,66]. However, contradictory to these findings, it was reported that subjects with high fetuin-A levels had a 4-fold increased risk for myocardial infarction and ischemic stroke compared to subjects with low fetuin-A levels [67]. In addition, individuals with previous myocardial infarction had significantly higher concentrations of fetuin-A than healthy controls [68]. A positive association of fetuin-A with arterial stiffness and increased intima–media thickness has been observed in healthy subjects and in patients with normal renal function [68,69,70].

In view of the above data, it seems that the relationship between circulating fetuin-A and CVD is complicated. The metabolic or cardiovascular risk is not only related to the serum levels of fetuin-A, but also to such factors such as renal function, concomitant disorders, medications and the type of serum assay used [15]. It is noteworthy that serum levels of fetuin-A may not clearly reflect the functionality of this hepatokine. Fetuin-A presents in circulation in two different forms in a soluble form in plasma as well as in colloidal fractions [71]. Phosphorylation of fetuin-A is needed for it be effective on insulin receptors [28,29]. Fetuin-A is fully phosphorylated only when it is in colloidal fractions [30]. Thus, simply measuring serum levels of fetuin-A may not be an accurate way to assess its functionality.

CVD is the most common cause of mortality in individuals with NAFLD. The latter is associated with an increased risk of incident CVD that is independent of the risk conferred by traditional cardiovascular risk factors (e.g., dyslipidemia, T2D and smoking) [13]. However, there is limited and conflicting data regarding the role of fetuin-A in the pathogenesis of increased CVD risk in NAFLD. Sato et al. investigated the relationship between circulating fetuin-A levels and liver/vessel fibrosis-related markers (platelet count, NAFLD fibrosis score and carotid intima–media thickness (cIMT)) in 295 subjects with NAFLD. Multivariate analysis revealed that fetuin-A concentration was a significant and independent determinant of platelet count, NAFLD fibrosis score and mean cIMT [44]. Our group investigated the relationship of circulating fetuin-A with markers of endothelial dysfunction (asymmetric dimethyl arginine (ADMA) and adiponectin) and cIMT in 115 patients with biopsy-confirmed NAFLD and 74 age-matched healthy subjects. Fetuin-A and ADMA levels were significantly higher and adiponectin level was significantly lower in the NAFLD group than the control group. In addition, the NAFLD group had greater cIMT levels than the controls. However, no difference was observed for fetuin-A, ADMA, adiponectin and cIMT between the two groups when the findings were adjusted for glucose, lipids and HOMA-IR index. In univariate analysis, fetuin-A was found to be positively associated with triglyceride, HOMA-IR, ADMA and cIMT values, and negatively associated with HDL-C and adiponectin. Multiple linear regression analysis showed that fetuin-A was independently associated with ADMA and cIMT levels [72]. In contrast, Ballestri et al. investigated fetuin-A levels in 70 subjects who underwent elective coronary angiography for suspected coronary artery disease (CAD) in a prospective, cross-sectional study. Twenty-four patients had no CAD (9 with and 15 without NAFLD) and 46 had CAD (20 with and 26 without NAFLD). Fetuin-A was significantly lower in patients with CAD compared to those without CAD. In addition, at multivariate analysis, they reported high fetuin-A levels to be independently associated with NAFLD and a lower risk of CAD [73]. Finally, Nascimbeni et al. investigated fetuin-A values and their relationship with symptomatic atherosclerosis in 149 patients with coronary artery disease (CAD) and peripheral arterial disease (PAD). Fetuin-A levels were positively associated with both CAD and NAFLD [74]. Studies investigating the relationship of fetuin-A with NAFLD-associated CVD risk are given in Table 2.

In light of these results, although the reason for the inconsistencies in the obtained data is not clear, various explanations have been suggested. It has been suggested that as a result of the detrimental effect of fetuin-A on insulin resistance and plasma lipid levels, it aggravates CVD in the initial period of the disease. However, in the later stages of the CVD, high fetuin-A levels have been observed to have a positive effect by preventing vascular calcification [75]. Therefore, it has been shown that the deficiency of fetuin-A, an inhibitor of vascular calcification, develops severe soft tissue and intravascular calcifications in animal studies with fetuin-A knockout mice [76]. On the other hand, as mentioned above, we observed a significant negative association of fetuin-A with HDL-C and adiponectin concentrations in our NAFLD cohort [72]. Since elevated levels of adiponectin and HDL-C are known to protect against atherosclerosis, we propose that the modulation of adiponectin and/or HDL-C by fetuin-A might be an important contributor in the pathogenesis of atherosclerosis and CVD in NAFLD. These data suggest that the relationship of fetuin-A with CVD is more complex than previously thought. Longitudinal studies with a greater number of subjects are needed to determine the contributory effects of fetuin-A on CVD risk in NAFLD.

## 4. Conclusions

NAFLD, considered a hepatic manifestation of MetS, independently increases the risk of developing both T2D and CVD. Hepatokines that are mainly secreted from the liver are known to affect glucose and lipid metabolism. They can also modulate inflammatory processes that in turn mediate the atherosclerotic process. Fetuin-A is a novel hepatokine and a pleotropic molecule with diverse and well-established proinflammatory and anti-inflammatory properties impacting a multitude of systems. As a proinflammatory compound, fetuin-A contributes to insulin resistance and is an important link between liver, adipose tissue and skeletal muscle. Although the significance of fetuin-A in NAFLD has been increasingly recognized, its pathogenetic role is still not completely understood. The relationship between circulating fetuin-A and CVD risk associated with NAFLD appears to be complex and in need of further research.

## Figures and Tables

**Table 1:** Studies investigating circulating fetuin-A levels in subjects with NAFLD.

Author (Reference)	Study Population	Diagnosis of NAFLD	Fetuin-A Levels in NAFLD (Compared to Controls)	Association of Fetuin-A with NAFLD
Lebensztejn et al. [38]	45 obese children with NAFLD and 30 controls	US	Higher	None
Reinehr et al. [39]	36 obese children with NAFLD and 14 controls	US	Higher	None
Ou HY et al. [40]	255 subjects with NAFLD and 255 controls	US	Higher	None
Huang et al. [41]	5219 middle-aged and elderly subjects	US	NE	Positive
Cui et al. [43]	79 subjects with NAFLD and 79 controls	US	Lower	Positive
Sato et al. [44]	295 subjects with NAFLD	US	NE	Negative
Thompson et al. [42]	78 subjects with NAFLD	CT	NE	None
Yilmaz et al. [45]	99 subjects with NAFLD and 75 controls	Liver bx	Higher	Positive
Haukeland et al. [46]	111 subjects with NAFLD and 131 controls	Liver bx	Higher	Positive
Ou et al. [47]	90 subjects with NAFLD and 90 controls	Liver bx	Higher	NE
Celebi et al. [48]	105 subjects with NAFLD	Liver bx	NE	None
Kahraman et al. [49]	108 morbidly obese subjects with NAFLD and 10 controls	Liver bx	NS	Negative
Rametta et al. [50]	137 subjects with NAFLD and 260 controls	Liver bx	Higher	Positive
Von Loeffelholz et al. [51]	58 subjects with NAFLD	Liver bx	NE	Positive
Pampanini et al. [52]	160 subjects with NAFLD and 23 controls	US and Liver bx	Higher	None

NAFLD: nonalcoholic fatty liver disease, Bx: biopsy, NE: not evaluated, NS: no significant difference, US: ultrasonography, CT: computed tomography.

**Table 2:** Studies investigating the relationship of fetuin-A with CVD in subjects with and without NAFLD.

Author (Reference)	Study Population	Study Design	CVD Risk Assessment	Association of Fetuin-A with CVD Risk
Jensen et al. [62]	3810 older subjects	Prospective	CVD event or CVD death	Negative
Laughlin et al. [63]	1688 women	Prospective	CVD event or CVD death	Negative
Sun et al. [64]	466 patients with IS	Prospective	IS	None
Caglar et al. [65]	198 nondiabetic patients with CKD	Retrospective	FMD and cIMT	Positive
Zhao et al. [75]	241 patients with T2D	Retrospective	Coronary angiography	Positive
Weikert et al. [67]	227 patients with MI and 168 patients with IS	Prospective	MI or IS	Positive
Vörös et al. [68]	171 patients with CVD and 81 controls	Cross-sectional	Biomarkers of ED	Positive
Mori et al. [70]	141 healthy subjects	Cross-sectional	Carotid arterial stiffness	Positive
Sato et al. [44]	295 subjects with NAFLD	Cross-sectional	cIMT	Negative
Dogru et al. [72]	115 subjects with NAFLD	Cross-sectional	Biomarkers of ED and cIMT	Positive
Ballestri et al. [73]	70 subjects with and without CVD	Cross-sectional	Coronary angiography	Negative
Nascimbeni et al. [74]	45 patients with CVD and 104 patients with PAD	Cross-sectional	CAD PAD	Positive

NAFLD: nonalcoholic fatty liver disease, CVD: cardiovascular disease, IS: ischemic stroke, CKD: chronic kidney disease, FMD: flow mediated dilation, cIMT: carotid intima–media thickness, T2D: type 2 diabetes, MI: myocardial infarction, ED: endothelial dysfunction, PAD: peripheral artery disease, CAD: coronary artery disease.

## Data Availability

Not applicable.
